# Editorial: Music and medicine: from basic science to clinical practice

**DOI:** 10.3389/fnins.2026.1792435

**Published:** 2026-03-23

**Authors:** Teresa Lesiuk, Indre V. Viskontas, Panagis Galiatsatos

**Affiliations:** 1 Music Therapy Department, Frost School of Music, University of Miami, University of Miami, Coral Gables, FL, United States; 2 University of San Francisco, San Francisco, CA, United States; 3 Johns Hopkins Medicine Sidney Kimmel Comprehensive Cancer Center, Baltimore, MD, United States

**Keywords:** music and clinical impact, music and development, music and neural systems, music-based interventions, sensorimotor responses to music

## 1 Introduction

The convergence of music and medicine is a fertile ground for innovative therapeutic interventions, now that the evidence base for how music can impact health has matured. Music-based interventions (MBIs) are not only effective in improving psychological wellbeing but also exert measurable physiological and neurological effects. Our call for papers for a Research Topic titled “*Music and Medicine: From Basic Science to Clinical Practice”* resulted in this Research Topic published in *Frontiers in Neuroscience: Auditory Cognitive Neuroscience*. We sought contributions from researchers studying the basic science of music, clinical applications of music-based interventions and theoretical models. This editorial distills the core themes, mechanisms, and clinical applications that emerged across the peer-reviewed collection of articles. The present *Frontiers Research Topic* and *ebook* underscore the breadth of music's influence via a peer-reviewed collection of 14 articles describing music processing as an underlying neural mechanism with implications for clinical treatment, as a structured therapeutic tool for neurological diseases and cancer, and as a stimulus for emotional, cognitive and psychological health.

## 2 Integrative framework: from neural mechanisms to clinical outcomes

Across these contributions, a coherent mechanistic framework emerges linking music's neural substrates to clinical outcomes. At the sensorimotor level, rhythmic auditory stimulation engages the sensorimotor cortex, supplementary motor area, and putamen through parallel cerebello-thalamo-cortical and striato-pallido-thalamocortical pathways, facilitating movement rehabilitation in Parkinson's disease (Harrison et al.) and informing how individual differences in motor and cognitive abilities predict synchronization success (Mudarris et al.). At the memory and default mode network level, familiar music activates the parahippocampal gyrus and medial prefrontal cortex, supporting autobiographical memory retrieval with implications for cognitive aging and early Alzheimer's intervention (Lesiuk et al.). Whole-brain network flexibility—the dynamic reorganization of functional communities—emerges as a key moderator: individuals with greater baseline flexibility derive stronger cognitive benefits from music creativity interventions (Wu-Chung et al.). At the autonomic and physiological level, music modulates heart rate variability and vagal tone, with singing interventions improving microvascular endothelial function in coronary artery disease (Bagherimohamadipour et al.) and music mindfulness acutely enhancing parasympathetic activity while altering frontotemporal neural oscillations in individuals with anxiety and depression (Ramirez et al.). These converging pathways—sensorimotor, limbic-memory, default mode, and autonomic—translate into clinical benefits spanning motor function, cognition, emotional resilience, and cardiovascular health, underscoring music's multifaceted therapeutic potential (see Table 1).

**Table 1:** Neural systems mapped to measures and outcomes in this Research Topic.

**Neural system**	**Key structures/measures**	**Clinical outcome**	**Source(s)**
Sensorimotor	Sensorimotor cortex, SMA, putamen, CTC/SPT pathways, cerebellum	Motor facilitation in PD; movement timing; gait rehabilitation	Harrison et al., Mudarris et al.
Limbic/memory	Parahippocampal gyrus, medial temporal lobe, mPFC	Autobiographical memory; cognitive aging; MCI intervention	Lesiuk et al.
Default mode network	mPFC, PCC/precuneus, whole-brain network flexibility/modularity	Global cognition in older adults; predictor of intervention response	Wu-Chung et al.
Autonomic/physiological	HRV (vagal tone), cortisol, alpha-amylase, endothelial function, frontotemporal EEG	Cardiovascular health (CAD); anxiety/depression; pain threshold; stress reduction	Bagherimohamadipour et al., Ramirez et al., Mallik et al.
Arousal/attention (bottom-up/top-down)	Vagal tone, effortful control, tempo-driven arousal modulation	Emotion regulation development (early childhood); resilience	Sena Moore et al., Heck et al.

Key themes that emerged are listed below.

### 2.1 Music as a structured therapeutic tool

A foundational theme is the importance of rigorously defining both the intervention, its putative mechanisms, and the measurable outcomes that can serve to assess it (Hanson-Abromeit). The therapeutic function of music plan (TFM) provides a framework for specifying the essential elements of music within MBIs, ensuring interventions are grounded in theory and tailored to individual needs. This approach is echoed in calls for improved reporting standards and scientific rigor, such as the NIH Music-based Intervention Toolkit and updated guidelines for MBIs (Robb et al.).

### 2.2 Music-use for developmental and cathartic release

#### 2.2.1 Early childhood

Music-based interventions in early childhood are designed to promote emotion regulation development. Theoretical models emphasize the role of tempo, physiological arousal, cognitive skills, and coregulation in fostering adaptive emotional skills through music experiences (Sena Moore et al.).

#### 2.2.2 Community healing

MBIs are also consistently shown to facilitate emotional regulation and resilience (Heck et al.) in adult populations. Live, relationship-centered music psychotherapy can effectively reduce depressive symptoms and increase resilience in vulnerable populations. Music-based assessment provides a culturally responsive, clinically meaningful, and cost-effective tool that deepens understanding of music's role in supporting wellbeing across diverse groups (Loewy et al.).

### 2.3 Neurological and cognitive mechanisms

#### 2.3.1 Parahippocampal–default mode network engagement: memory and aging

Music-evoked autobiographical memories (MEAMs) offer a window into how familiar music engages memory-related neural circuitry. Lesiuk et al. demonstrate that when older adults listen to personally familiar music, *fractional amplitude of low frequency fluctuations (fALFF) increases in the right parahippocampal gyrus*—a region critical for contextual memory associations and one that interfaces with the default mode network during self-referential processing. This activation occurs with minimal retrieval effort, suggesting that music provides privileged access to long-term memory systems. Because the parahippocampal cortex is among the earliest regions affected in prodromal Alzheimer's disease, these findings point toward MEAMs as a potential non-pharmacological intervention for populations with mild cognitive impairment, leveraging intact musical memory circuits to stimulate vulnerable memory networks.

#### 2.3.2 Whole-brain network dynamics: cognitive change and individual differences

Beyond regional activation, the brain's capacity to dynamically reorganize functional networks—termed *network flexibility*—emerges as a critical moderator of music's cognitive benefits. Wu-Chung et al. show that a 6-week music creativity intervention improved global cognition, but *only among individuals with higher baseline whole-brain network flexibility*. This finding suggests that the ability to fluidly reconfigure neural networks—previously linked to executive functioning and adaptive learning—may be a prerequisite for extracting cognitive benefits from music engagement. Clinically, this finding implies that neuroimaging-based indices of network dynamics could help identify which older adults are most likely to benefit from music-based cognitive interventions, enabling more personalized therapeutic targeting.

#### 2.3.3 Sensorimotor networks: rhythmic synchronization and motor rehabilitation

Rhythmic auditory stimulation supports movement through tight coupling between auditory and motor systems. Harrison et al. used fMRI to examine both external (listening to music) and internal (mental singing) cues during finger tapping in people with Parkinson's disease and healthy controls. Both cue types activated *sensorimotor cortex, supplementary motor areas, and putamen*, with external cues producing greater activation in auditory cortex, whereas internal cues showed preferential recruitment of *cerebellar* networks. Critically, no differences emerged between patient and control groups, suggesting that both *cerebello-thalamo-cortical (CTC)* and *striato-pallido-thalamocortical (SPT)* pathways remain functional and can be harnessed therapeutically. This dual-pathway model implies that music-based therapies may leverage remaining function in degenerating basal ganglia structures while also activating compensatory cerebellar routes—a finding with direct implications for gait rehabilitation in Parkinson's disease.

Complementing this neuroimaging work, Mudarris et al. examined how individual differences in cognitive and motor abilities predict sensorimotor synchronization during finger tapping. They found that *cognitive inhibition and fine/gross motor abilities* significantly predicted tapping force and timing consistency, highlighting that the efficacy of rhythmic auditory stimulation depends on baseline capacities. Together, these studies underscore the importance of tailoring music-based motor interventions to individual neurocognitive profiles.

#### 2.3.4 Neurochemistry, pain and stress modulation

At the neurochemical level, Mallik et al. demonstrate that group singing in Parkinson's disease patients *reduces cortisol and alpha-amylase*—stress-related biomarkers linked to the hypothalamic-pituitary-adrenal axis—which correlate with increases in pain threshold. This neuroendocrine pathway provides a mechanistic account of how communal music-making may alleviate pain and improve quality of life beyond subjective report.

### 2.4 Music-based interventions in clinical populations

#### 2.4.1 Parkinson's disease

Both external (listening to music) and internal (mental singing) musical cues activate sensorimotor and auditory brain regions, facilitating movement in people with Parkinson's disease and healthy controls (Harrison et al.) These cues utilize parallel neural pathways, implying that music-based therapies may leverage remaining function in degenerating areas and activate alternative routes for sensorimotor activity.

#### 2.4.2 Disorders of consciousness

Musical Sensory Orientation Training (MSOT) is presented as a non-invasive technique to improve consciousness and cognitive function in patients with disorders of consciousness (Gu et al.) MSOT demonstrated feasibility and efficacy in clinical settings, with significant improvements in communication and arousal levels.

#### 2.4.3 Group singing and neurochemistry

Group singing in Parkinson's disease patients is associated with reductions in cortisol and alpha amylase, which are linked to increases in pain threshold. This finding suggests one neurochemical pathway by which music can alleviate pain and improve quality of life in those with PD (Mallik et al.).

#### 2.4.4 Cardiovascular health

Singing interventions, both live and video-based, improve microvascular endothelial function and heart rate variability in older adults with coronary artery disease. These physiological changes are comparable to those observed with exercise, highlighting music's potential as a non-pharmacological adjunct for cardiovascular health (Bagherimohamadipour et al.) especially in adults who might be physically-limited in terms of their ability to exercise.

#### 2.4.5 Oncology

Active music engagement (AME) is shown to mitigate traumatic stress symptoms in parents and children undergoing cancer treatment. Sociodemographic factors and risk profiles help identify families who may benefit most from music therapy interventions (Robb et al.).

### 2.5 Music mindfulness and autonomic regulation

Music mindfulness, which combines music listening with mindfulness activities, acutely enhances heart rate variability and alters neural activity, effectively targeting the autonomic nervous system and brain networks involved in goal-directed behavior and emotional regulation. These effects are beneficial for treating symptoms of anxiety and depression, with live sessions also increasing social connection (Ramirez et al.).

## 3 Conclusion

Taken together, these reports collectively demonstrate that music is a multifaceted therapeutic agent in medicine, with applications ranging from mental health and neurological rehabilitation to cardiovascular and oncological care. Its efficacy is supported by neurobiological, physiological, and psychological evidence, and its integration into clinical practice is guided by evolving scientific standards, including careful monitoring at the level of the individual, and, when appropriate, personalization of the intervention. Music's ability to engage brain networks, regulate emotion, and foster resilience underscores its value as both a preventive and rehabilitative tool in modern healthcare. Research Topics like this one that bring together a diverse set of findings enable researchers to build mechanistic frameworks that will lead to more efficient translation in the hands of practitioners.

